# Novel mutations in *TPM2* and *PIEZO2* are responsible for distal arthrogryposis (DA) 2B and mild DA in two Chinese families

**DOI:** 10.1186/s12881-018-0692-8

**Published:** 2018-10-03

**Authors:** Shan Li, Yi You, Jinsong Gao, Bin Mao, Yixuan Cao, Xiuli Zhao, Xue Zhang

**Affiliations:** 1 Department of Medical Genetics, Institute of Basic Medical Sciences, Chinese Academy of Medical Sciences & School of Basic Medicine, Peking Union Medical College, 5 Dong Dan San Tiao, Dong Cheng District, Beijing, 100005 China; 2 Department of Obstetrics and Gynecology, Peking Union Medical College Hospital, Beijing, 100730 China

**Keywords:** Distal arthrogryposis, *TPM2*, *PIEZO2*, Novel mutation, Genotype–phenotype

## Abstract

**Background:**

Distal arthrogryposis (DA) is a group of clinically and genetically heterogeneous disorders that involve multiple congenital limb contractures and comprise at least 10 clinical subtypes. Here, we describe our findings in two Chinese families: Family 1 with DA2B (MIM 601680) and Family 2 with mild DA.

**Methods:**

To map the disease locus, two-point linkage analysis was performed with microsatellite markers closed to *TPM2*, *TNNI2/TNNT3* and *TNNC2.* In Family 1, a positive LOD (logarithm of odds) score was only obtained at the microsatellite marker close to *TPM2* and mutation screening was performed using direct sequencing of *TPM2* in the proband. In Family 2, for the LOD score that did not favor linkage to any markers, whole-exome sequencing (WES) was performed on the proband. PCR–restriction fragment length polymorphism (RFLP) and bioinformatics analysis were then applied to identify the pathogenic mutations in two families. In order to correlate genotype with phenotype in DA, retrospective analyses of phenotypic features according to the *TPM2* and *PIEZO2* mutation spectrums were carried out.

**Results:**

A heterozygous missense mutation c.308A > G (p.Q103R) in *TPM2* in Family 1, and a novel variation c.8153G > A (p.R2718Q) in *PIEZO2* in Family 2 were identified. Each of the two novel variants was co-segregated with the DA manifestations in the corresponding family. Bioinformatics analysis from several tools supported the pathogenicity of the mutations. Furthermore, our study suggests that there is no relation between the types or locations of *TPM2* mutations and the clinical characteristics, and that different inheritance modes and mutation types concerning *PIEZO2* cause distinct clinical manifestations.

**Conclusions:**

We report two novel mutations within *TPM2* and *PIEZO2* responsible for DA2B and mild DA in two Chinese families, respectively. Our study expands the spectrum of causal mutations in the *TPM2* and *PIEZO2* genes.

**Electronic supplementary material:**

The online version of this article (10.1186/s12881-018-0692-8) contains supplementary material, which is available to authorized users.

## Background

Distal arthrogryposis (DA) is a group of rare autosomal-dominant limb disorders that involve congenital contractures of two or more body areas without primary neurologic or muscular disease [1]. DA is clinically and genetically heterogeneous and comprises more than 10 clinical subtypes (1, 2A, 2B, 3–10) based on additional phenotypic features [2]. The most common subtypes are DA1 and DA2B [3]. The hallmarks of DA1 include camptodactyly and clubfoot, without additional abnormalities [4]. Patients with DA2B typically present severe camptodactyly, triangular face, prominent nasolabial folds, attached earlobes, downward-slanting palpebral fissures, small mouth, and prominent chin [5]. DA3 (Gordon syndrome, MIM 114300) is distinguished from other DA by short stature and cleft palate [6]. A small subset of DA5 (MIM 615065) is known by the presence of ocular abnormalities, typically ptosis, ophthalmoplegia, and/or strabismus, in addition to contractures of the skeletal muscles [2]. To date, mutations in *TPM2*, *TNNI2*, *TNNT3*, and *MYH3* which encode components of the contractile apparatus of fast-twitch myofibers or embryonic myosin, have been implicated in DA1 and DA2B [5, 7]. The *PIEZO2* gene encodes a mechanically activated cation channel, and the mutations of *PIEZO2* have been reported in DA3, DA5 and Marden-Walker syndrome MWKS [8].

In the current study, we recruited two families affected by DA2B and mild DA, and conducted two-point linkage analysis and Sanger sequencing or/and whole-exome sequencing (WES) to identify pathogenic mutations. Our findings indicated that a novel mutation in *TPM2* was responsible for DA2B in Family 1, and that a missense mutation in *PIEZO2* was the etiology of mild arthrogryposis in Family 2. This is the first finding that *TPM2* and *PIEZO2* are associated with DA in Chinese population, which provided new clues for the correlation between genotype and phenotype in DA.

## Methods

### Participants collection and isolation of genomic DNA

**Fig. 1:**
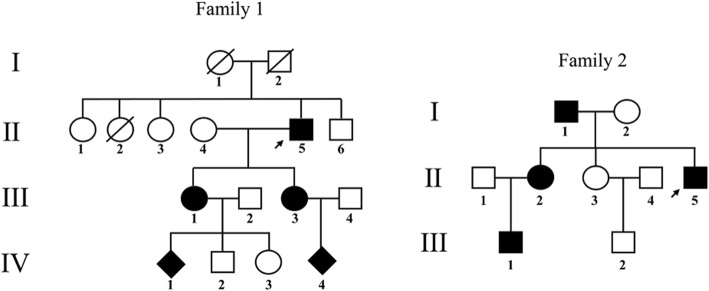
Pedigrees of the two families with DA2B (Family 1) and mild DA1 (Family 2). Arrows indicate the probands in each family

Two Chinese pedigrees with autosomal dominant hereditary DA were collected (Fig. 1). Careful examinations and clinical record reviews were performed for each patient to confirm the original diagnosis. Peripheral blood or cord blood samples were obtained from all participants. Genomic DNA was isolated from blood samples by the standard method of proteinase K and phenol/chloroform extraction [9]. All participants provided informed consent, and this study was approved by the ethics committee of the Peking Union Medical College Institutional Review Board.

### Linkage analysis

Using the UCSC Genome Browser (http://genome.ucsc.edu), three microsatellite repeat sequences near *TPM2* (chr9: 35,681,993-35,690,056), *TNNI2* (chr11: 1,840,202-1,841,680)/*TNNT3* (chr11: 1,919,569-1,938,706), and *TNNC2* (chr20: 45,823,216-45,827,314) were selected as markers for two-point linkage analysis (Additional file 1: Table S1). PCR products were separated by conventional 8% polyacrylamide gel electrophoresis. Two-point linkage analysis was carried out using the MLINK program in the LINKAGE software package (version 5.2) [10]. The assumptions of the analysis were autosomal dominant inheritance, penetrance of 100%, a disease allele frequency of 0.0001, and equal recombination frequencies for males and females.

### Whole-exome sequencing

In Family 2, exome capture was performed using an Agilent SureSelect Human All Exon kit (Agilent Technologies, Wilmington, DE), according to the manufacturer’s instructions. DNA was broken into fragments of 180–280 bp using an ultrasonoscope. A sequencing-capture DNA library was prepared by means of a HiSeq 2000 platform (Illumina, San Diego, CA). Collection of primary data, including error assessment and base calling, was accomplished with an Illumina Pipeline (version 1.3.4) [11]. Sequencing reads were aligned to the human reference genome sequence from the UCSC database. Variants were filtered by the Single Nucleotide Polymorphism Database (dbSNPs; https://www.ncbi.nlm.nih.gov/projects/SNP/) and the 1000 Genomes database (www.internationalgenome.org). Reported disease-causing mutations were confirmed based on the Human Gene Mutation Database at the Institute of Medical Genetics in Cardiff (HGMD, http://www.hgmd.cf.ac.uk/ac/index.php) or previous literature. Functional domain was predicted by the SMART database (http://smart.embl-heidelberg.de).

### Validation of mutations

For Family 1, *TPM2* (the primary candidate gene) was amplified by PCR using 5 primer pairs (E1–2, E3–6, E7–9, E10, and E11; Additional file 1: Table S1). To validate the mutation identified in proband, co-segregation analysis in the family was applied by PCR-RFLP. PCR fragments amplified by primer E3F (forward) and In-E4R (reverse), which were digested by the restriction endonuclease *Sma* I, were separated by 2% agarose gel electrophoresis.

For Family 2, PCR and Sanger DNA sequencing were performed to verify the variant in *PIEZO2* (Additional file 1: Table S1). Amplicons from all available family members were analyzed by RFLP using *Taq* I and 8% polyacrylamide gel electrophoresis.

### Bioinformatics analysis

The amino acid sequences of the tropomyosin beta chain (TPM2; NP_001288156.1), component 2 of the piezo-type mechanosensitive ion channel (PIEZO2; NP_071351.2) and their homologues of 10 animal species were obtained from the NCBI (National Center for Biotechnology Information) protein database in FASTA format. Multiple-sequence alignment and conservation analysis were performed with MEGA software (version 7; Institute for Genomics and Evolutionary Medicine, Temple University, Philadelphia, PA). The pathogenicity of missense variants was predicted by PolyPhen-2 (http://genetics.bwh.harvard.edu/pph2), Scale-Invariant Feature Transform (SIFT; http://sift.jcvi.org), Protein Variation Effect Analyzer (PROVEAN; http://provean.jcvi.org/seq_submit.php), Mutation Taster (http://www.mutationtaster.org) and M-CAP (http://bejerano.stanford.edu/mcap). The mutant proteins translated from the missense variants in *TPM2* and *PIEZO2* were assessed using the crystal structure of contractile protein at 35 Å resolution (PDB code 2W4U) and metal transport protein at 4.8 Å resolution (PDB code 3JAC), respectively. SWISS-MODEL was used to model the amino acid changes in 3D structure (SWISS-MODEL; http://swissmodel.expasy.org/). Hydrogen (H)-bonding interactions with neighboring residues were visualized with the Swiss-Pdb Viewer (version 4.10; Swiss Institute for Bioinformatics, Lausanne, Switzerland).

## Results

### Clinical evaluation

**Fig. 2:**
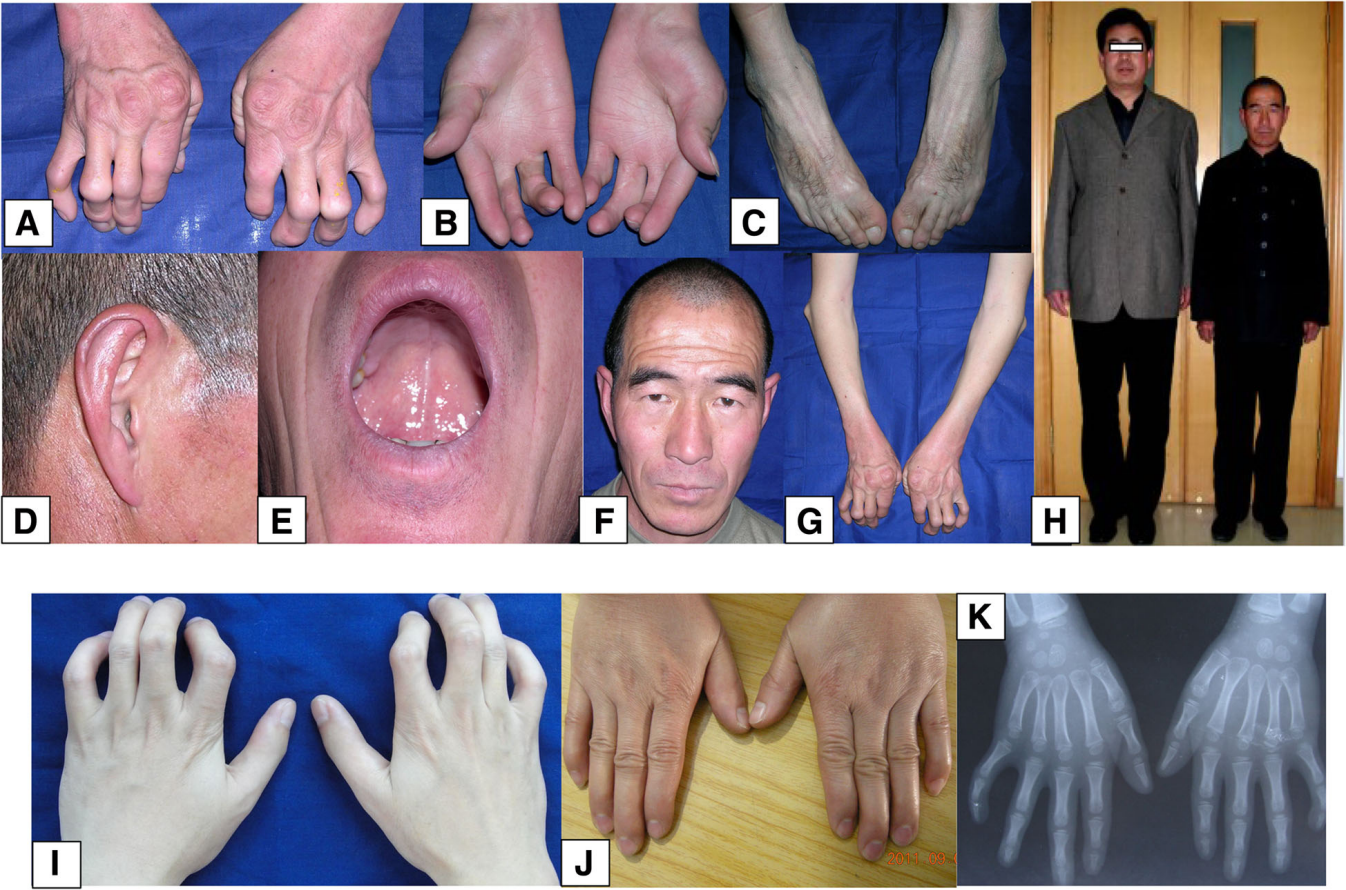
Clinical features of patients in Family 1 with DA2B (**a**-**h**) and Family 2 with mild DA (**i**-**k**). **a** Ulnar deviation and contractures in patient II5. **b** Camptodactyly and adducted thumbs in patient III1. **c** Flexed toes and talipes equinovarus in patient II5. **d** Dysplastic ear and attached ear lobes in patient II5. **e** Small mouth with limited opening in patient II5. **f** Downward-slanting palpebral fissures, a small chin, and deep folds in the nasolabial area and forehead in patient II5. **g** Flexion contracture yielding stiff elbows in patient II5. **h** Short stature of patient II5 (unaffected II6 [left], 178 cm; patient II5 [right], 156.5 cm). **i** Camptodactyly and ulnar deviation in patient II5. **j** Mild contractures of the fingers in patient II2. **k** X-ray findings of patient III1 in 5 years old indicate camptodactyly and ulnar deviation

Family 1 was a four-generation pedigree involving DA of variable severity (Fig. 1). Four patients and a fetus of 22-week gestational age were recruited for phenotypic evaluation by clinical examination or by administration of a questionnaire. The proband (II5) was a 59-year-old male with bilateral and symmetric congenital contractures of the distal limbs, including severe ulnar deviation, camptodactyly, adducted thumbs and overlapping fingers (Fig. 2a, g). Short stature and minor facial anomalies, including a triangular face, downward-slanting palpebral fissures, and a small mouth, were also noted in the proband, but not in his two affected daughters (III1 and III3) (Fig. 2d-h). The proband had more severe phenotype than his two affected daughters did (Fig. 2b-c), indicating phenotypic variability in the family. Patient III1 was pregnant, whose fetus was diagnosed to have phenotype of DA on ultrasound examination. In particular, the ultrasound results indicated extended wrists, clenched fists, and bilateral club feet (Additional file 2: Figure S1). According to the clinical characteristic of the patients mentioned above, we concluded that this family was affected by D2AB (Table 1).

**Table 1:** Clinical phenotypes of patients with TPM2 and PIEZO2 gene mutations in two families

	Family 1			Family 2			
	II-5	III-1	III-3	II-5	I -1	II-2	III-1
Age/Sex	59/M	35/F	32/F	37/M	65/M	42/F	11/M
Decreased facial expression	–	–	–	–	–	–	–
Ptosis/Limited ocular motility	+	–	–	–	–	–	–
Ear deformity	–	–	–	–	–	–	–
Small pursed mouth	+	–	–	–	–	–	–
Trismus	–	–	–	–	–	–	–
Cleft palate	–	–	–	–	–	–	–
Nasolabial folds	+	–	–	–	–	–	–
Triangularly shaped face	+	–	–	–	–	–	–
Finger contractures	+	+	+	+	+	+	+
Camptodactyly	+	+	+	+	+	+	+
Elbow contractures	+	–	–	–	–	–	–
Limited wrist extension	+	–	–	–	–	–	–
Asymmetric legs/feet	–	–	–	–	–	–	–
Clubfeet	+	+	+	+	+	+	+
Scoliosis	–	–	–	–	–	–	–
Short stature	+	–	–	+	+	+	ND
Muscle weakness	–	–	ND	–	–	–	–
Sensorineural hearing loss	–	–	–	–	–	–	–
Pain problems	–	–	–	–	–	–	–
Surgical operations	+	+	ND	–	–	–	–
Additional symptoms	–	–	ND	–	–	–	–
Radiographs	–	–	–	+	–	–	+
DA classification	DA2B			DA1			
Disease-causing mutation	*TPM2*: c.308A > G			*PIEZO2:*c.8153G > A			

+,present;-, absent; *NA* information not available, *ND* not determined

Family 2 was a three-generation pedigree with mild DA, and including four affected individuals and five unaffected family members (Fig. 1). The proband (II5) was a 37-year-old man, with congenitally bilateral-symmetric contractures in fingers 2–5 (Fig. 2i). The other three affected family members had mild contractures restricted to the distal phalanges (Fig. 2j-k). All the affected individuals presented variable arthrogryposis without other manifestations of DA3, DA2B, or DA5 such as cleft palate or ocular/facial abnormalities (Table 1).

### Linkage analysis

**Fig. 3:**
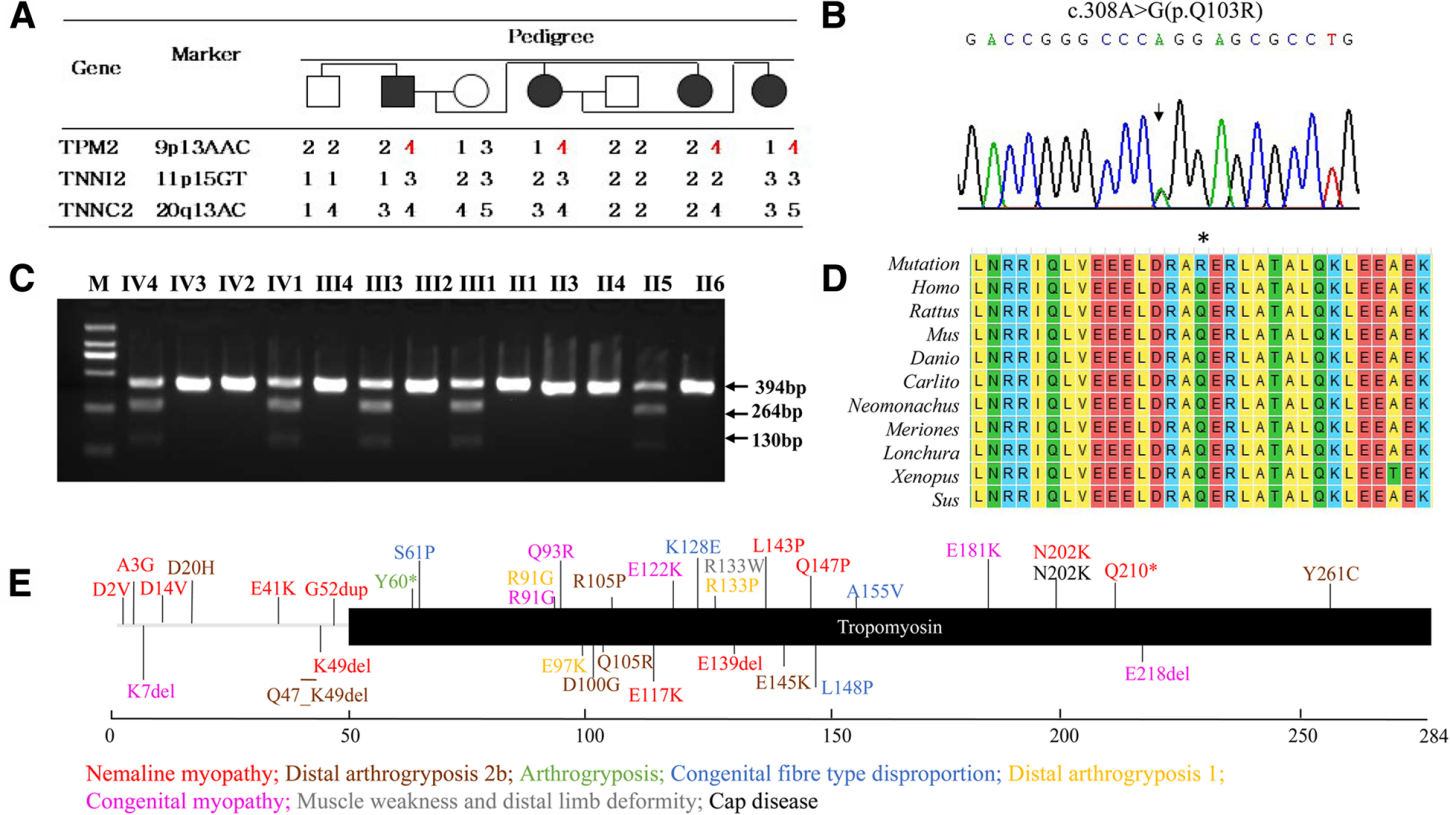
Identification of a missense mutation in *TPM2* in Family 1. **a** Genetic linkage analysis was carried out with 3 microsatellites, and *TPM2* was identified as the candidate gene. **b** Sequencing results indicate the heterozygous mutation c.308A > G in exon 3 of *TPM2*. **c** DNA fragments from the affected individuals (II5, III1, III3, IV1, and IV4). Three fragments were separated by electrophoresis (394 bp, 264 bp, and 130 bp). **d** Multiple-species sequence alignment shows the evolutionary conservation of position p.Q103 in TPM2. **e** The schematic of *TPM2* mutation spectrum on functional module. Mutation-related diseases are exhibited in different literal colors

For Family 1, two-point linkage analysis yielded a positive LOD score for 9p13AAC near *TPM2*, indicating linkage of the loci (Additional file 3: Table S2 and Fig. 3a). LOD scores for the other two markers near *TNNI2*/*TNNT3* and *TNNC2* did not favor linkage. Therefore, *TMP2* was regarded as the candidate gene for Family 1. For Family 2, LOD scores indicated no linkage for any of the three locations.

### Identification of a missense mutation in *TPM2* in family 1

Sanger sequencing of the proband indicated the presence of a heterozygous missense mutation c.308A > G in exon 3 (Fig. 3b). This mutation led to the substitution of glutamine for arginine at position 103 of the amino acid sequence (Q103R) and yielded a novel *Sma* I restriction site in the mutant *TPM2* gene. PCR products digested by *Sma* I were separated by 2% agarose gel electrophoresis. All unaffected individuals had the expected 394 bp fragments, whereas all affected members had 394 bp, 264 bp and 130 bp fragments (Fig. 3c).

### Identification of a missense mutation in *PIEZO2* in family 2

**Fig. 4:**
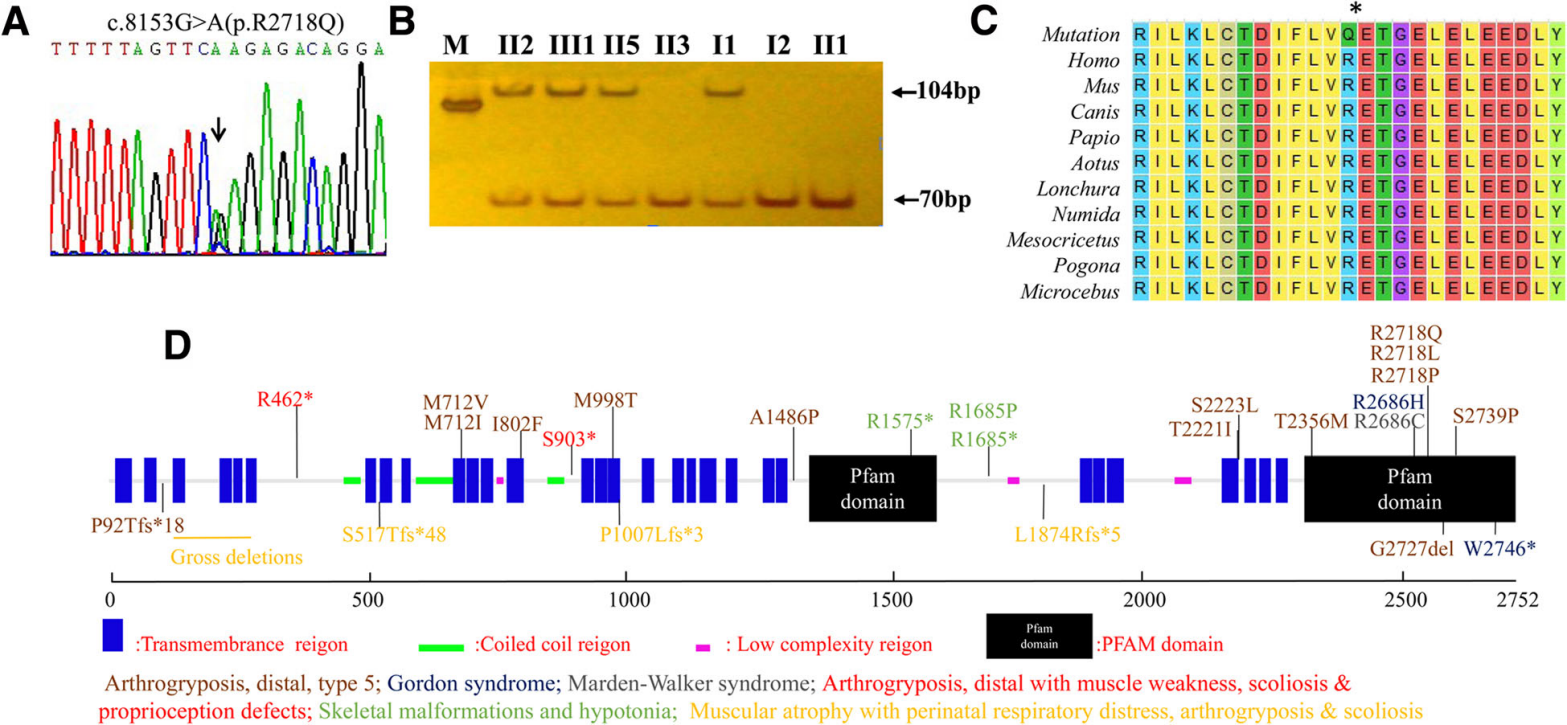
Identification of a missense mutation in *PIEZO2* in affected members of Family 2. **a** The novel mutation c.8153G > A (p.R2718Q) in *PIEZO2* verified by Sanger sequencing. **b** PCR-RFLP findings with *Taq* I indicate two DNA fragments in affected patients (II2, III1, II5, and I1; 104 bp and 70 bp) but not in unaffected family members. **c** Multiple-species sequence alignment shows the evolutionary conservation of position p.R718 in *PIEZO2*. **d** The schematic of *PIEZO2* mutation spectrum on functional module. Mutation-related diseases are exhibited in different literal colors

On average, 90% coverage was achieved for exome regions from genomic DNA samples (II5) of Family 2, and the sequencing depth exceeded 100×. A novel heterozygous variant was found in exon 52 of *PIEZO2* in the proband. This mutation resulted in the substitution of glutamine to arginine (p.R2718Q) and abrogated the restriction site recognized by *Taq* I (Fig. 4a). Co-segregation in members of Family 2 was observed in PCR-RFLP results. The mutant-allele amplicon could not be digested by *Taq* I and yielded a 104 bp fragment, whereas the wild-type-allele amplicon was digested by *Taq* I to produce 70 bp and 34 bp fragments. All samples from affected members of Family 2 (II2, III1, II5, and I1) yielded three fragments (104 bp, 70 bp, and 34 bp); in contrast, all samples from unaffected members produced only two fragments (70 bp and 34 bp) (Fig. 4b).

### Results of bioinformatics analysis

Conservation analysis of p.Q103 in TPM2 and p.R2718 in PIEZO2 among 10 species indicated that the two amino acid sites are highly conserved (Figs. 3d and 4c). There is no record about the frequency of the two mutations in ExAC browser, esp6500si, 1000 Genome, dbSNP, gnomAD browser, etc. Mutations p.Q103R in *TPM2* and p.R2718Q in *PIEZO2* were predicted to be potentially damaging by bioinformatics analysis from softwares SIFT, Polyphen2, MutationTaster, M-CAP, and PROVEAN programs. These prediction programs yielded similar outcomes regarding pathogenicity except that Polyphen2 predicted p.Q103R a benign mutation. Therefore, substitution of wild-type residues might change the biological function. (Table 2).

**Table 2:** Predictions of functional effects for two mutations in this study

	SIFT	Polyphen-2	MutationTaster	M-CAP	PROVEAN
TPM2:c.308A > G(p.Q103R)	Damaging	Benign	Disease causing	Possibly Pathogenic	Deleterious
score	0	0.06	–	0.349	−3.13
PIEZO2:c.8153G > A (p.R2718Q)	Damaging	Probably Damaging	Disease causing	Possibly Pathogenic	Deleterious
score	0	1	–	0.821	−3.35

**Fig. 5:**
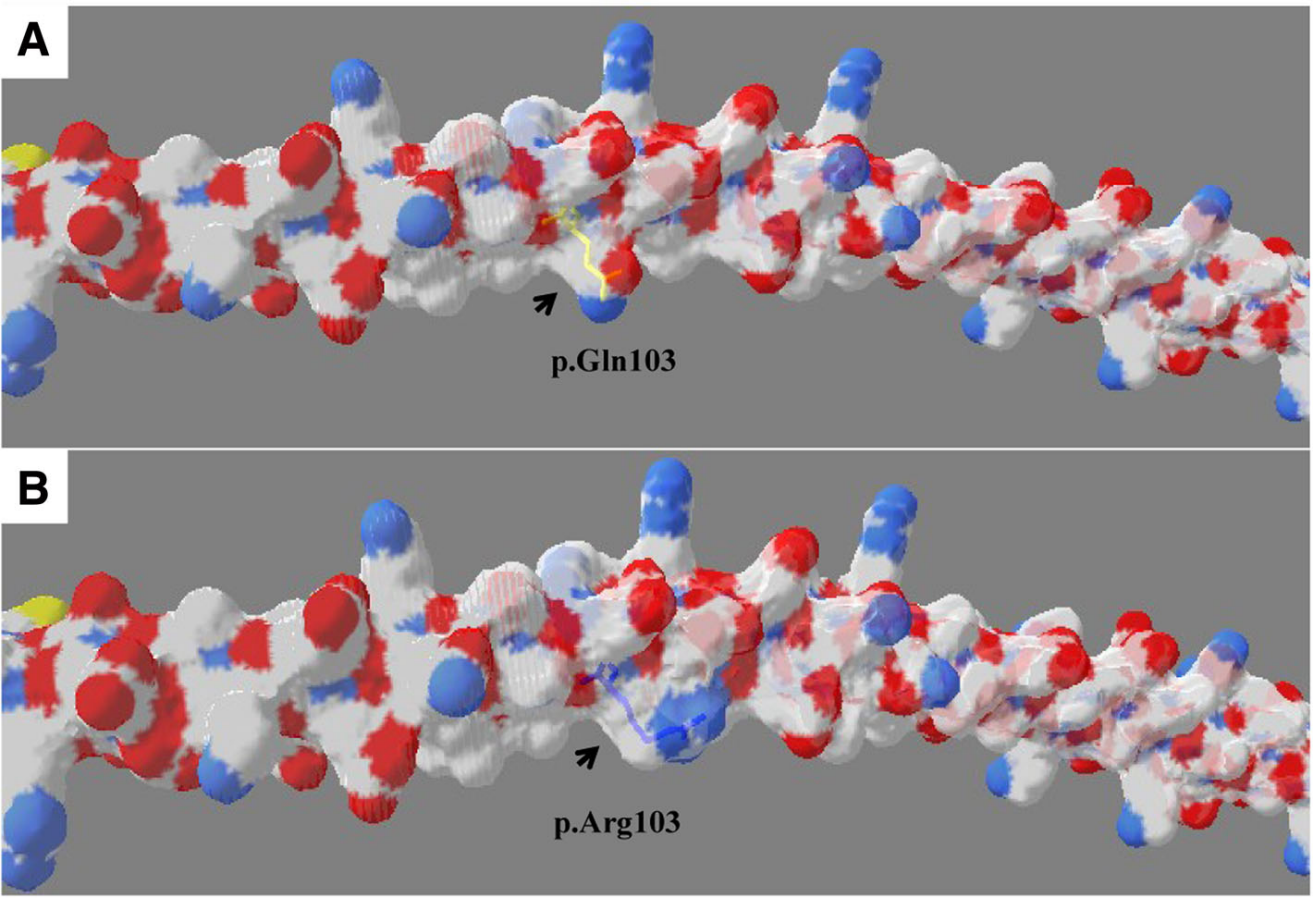
SWISS-MODEL prediction of the p.Q103R mutation in tropomyosin. **a** The 3-dimensional structure of wild-type tropomyosin with position 103 indicated by a black arrow. Nearby acidic (red) and basic (blue) surface properties are illustrated with the neutral glutamine residue shown as a yellow line. **b** Acidic surface properties are destroyed with the substitution of basic arginine for glutamine. The substitution is depicted as a blue line

**Fig. 6:**
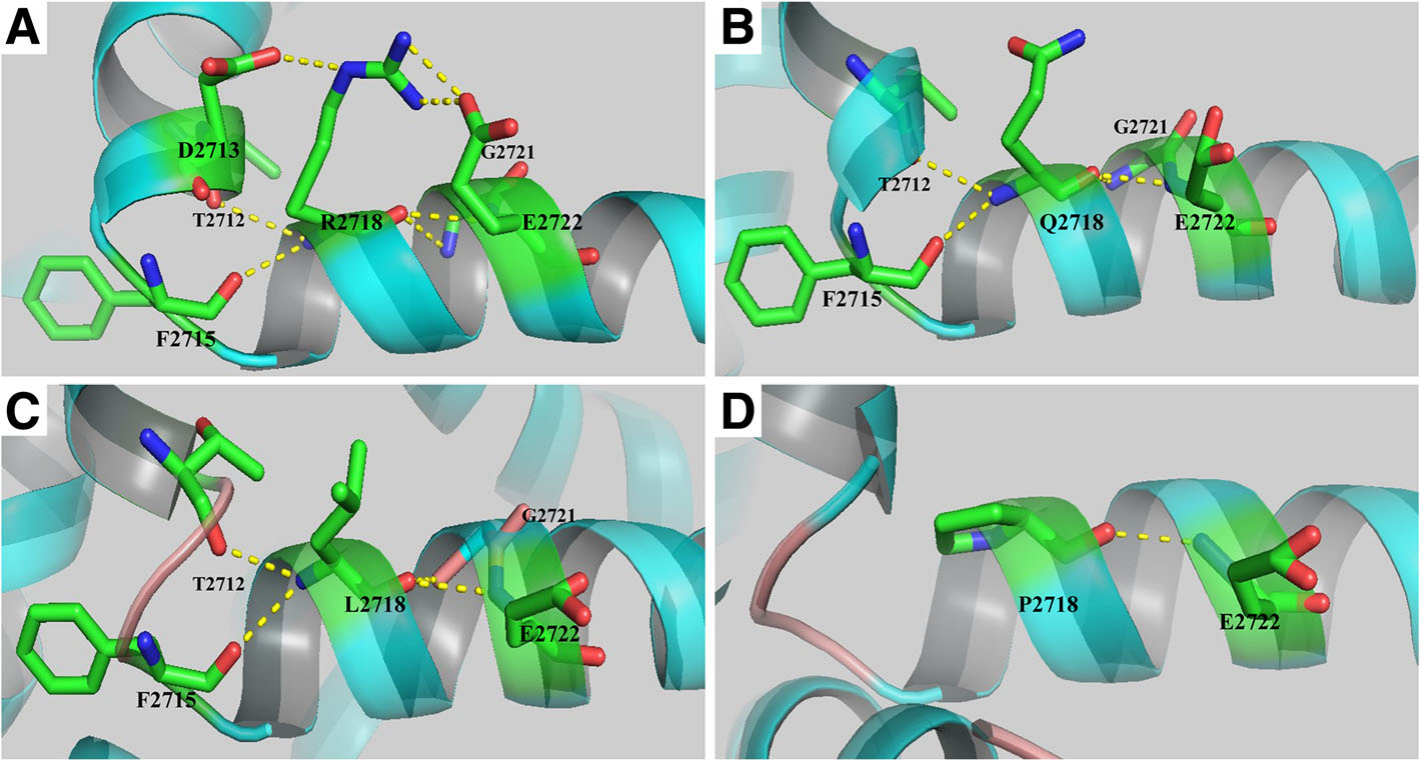
Three substitutions in position 2718 of *PIEZO2* are simulated by means of SWISS-MODEL and are represented with Ribbon. **a** The wild-type amino acid at position 2718 is depicted, with H-bonds shown by yellow dashed lines. **b** The p.R2718Q substitution maintains the most parts of H-bonding interactions. **c** Leucine tends to lose H-bond with D2713, and the nearby α-helical structure is disrupted. **d** Substitution with proline eliminates most of the H-bonds with neighboring amino acids

According to the SWISS-MODEL prediction, substitution of a neutral amino acid (glutamine) to a basic amino acid (arginine) at position 103 of tropomyosin would likely influence the conformation and charge properties of the protein. In addition, the presence of arginine may alter the acidic surface properties of the protein and could potentially impair its molecular function (Fig. 5). Three substitutions in position 2718 of component 2 of the piezo-type mechanosensitive ion channel were simulated by SWISS-MODEL and represented with Ribbon. This simulation predicted that R2718 interacted via H-bonding with residues D2713, T2712, F2715, G2721 and E2722. Substitution of p.R2718Q destroyed the H-bonding between R2718 and D2713 (Fig. 6b). This change could interfere with the protein function by conformational alteration.

### Genotype–phenotype correlations

According to the mutation of *TPM2* in this study and retrospective analysis of other related studies, we found there is no any significant clear correlation between types or location of *TPM2* mutations and clinical manifestations (Fig. 3e).

The patients of Family 2 carrying the mutation p.R2718Q in *PIEZO2* have mild arthrogryposis without other manifestations. It was interesting to note that the mutation p.R2718Q was located at the same site as two previously reported mutations (p.R2718P and p.R2718L). Although all the three mutations located at the same site, mutation p.R2718P and p.R2718L led to more severe phenotypes. We constructed three-dimensional models of the mutated proteins to investigate the possible molecular pathogenicity. We found that substitution of leucine tended to lose H-bond with D2713, and to disrupt the structure of neighbor α-helical (Fig. 6c). On the contrary, substitution of proline did not affect the α-helix, but destroyed the connection between H-bonds to T2712, D2713 and F2715 (Fig. 6d). We noticed that substitution of glutamine was prone to maintain the original H-bonds and keep the original molecular conformation (Fig. 6b), which enlighten the association between genotypes and phenotypes.

Further, analysis indicated that DA3, DA5, MWKS patients with dominant inheritance carried heterozygous mutations which are mainly localized at the C-terminal of PIEZO2*,* and that recessive inherited *PIEZO2*-related disorders are all caused by biallelic truncating mutations in *PIEZO2* (Table 3) (Fig. 4d).

**Table 3:** List of PIEZO2 mutations reported in the literatures

Family No.	cDNA Change	Predicted Protein Alteration	Inheritance Model	Origin	References
Arthrogryposis, distal, type 5					
1	c.2136G > A	p.M712I	AD	UK	Harris (2017) Orphanet J Rare Dis 12, 151 [24]
2	c.2134A > G	p.M712 V	AD	NA	McMillin (2014) Am J Hum Genet 94, 734 [8]
3	c.2404A > T	p.I802F	AD	USA	Coste (2013) Proc Natl Acad Sci U S A 110, 4667 [23]
4	c.2993 T > C	p.M998 T	AD	NA	McMillin (2014) Am J Hum Genet 94, 734 [8]
5	c.4456G > C	p.A1486P	AD	Japan	Okubo (2015) Am J Med Genet A 167, 1100 [22]
6	c.6662C > T	p.T2221I	AD	NA	McMillin (2014) Am J Hum Genet 94, 734 [8]
7	c.6668C > T	p.S2223 L	AD	NA	McMillin (2014) Am J Hum Genet 94, 734 [8]
8	c.7067C > T	p.T2356 M	AD	NA	McMillin (2014) Am J Hum Genet 94, 734 [8]
9	c.8153G > T	p.R2718L	AD	NA	McMillin (2014) Am J Hum Genet 94, 734 [8]
10	c.8153G > C	p.R2718P	AD	NA	McMillin (2014) Am J Hum Genet 94, 734 [8]
11	c.8215 T > C	p.S2739P	AD	NA	McMillin (2014) Am J Hum Genet 94, 734 [8]
12	c.273_279delACCTGGC	P92Tfs*18	AD	Saudi Arabia	Alfares (2017) Mol Genet Metab 121, 91 [28]
					Monies (2017) Hum Genet 136: 921 [Additional phenotype] [29]
13	c.8181_8183delAGA	p.G2727del	AD	USA	Coste (2013) Proc Natl Acad Sci U S A 110, 4667 [23]
14	c.8208delA	p.Y2737Ifs*7	AD	NA	McMillin (2014) Am J Hum Genet 94, 734 [8]
15	c.8153G > A	p.R2718Q	AD	China	Current study
Marden-Walker syndrome					
16	c.8056C > T	p.R2686C	AD	NA	McMillin (2014) Am J Hum Genet 94, 734 [8]
Gordon syndrome					
17	c.8057G > A	p.R2686H	AD	NA	McMillin (2014) Am J Hum Genet 94, 734 [8]
18	c.8057G > A	p.R2686H	AD	Germany	Alisch (2017) Am J Med Genet A 173: 254 [Additional case report] [30]
19	c.8238_8245delGACTAGAG	p.W2746*	AD	NA	McMillin (2014) Am J Hum Genet 94, 734 [8]
Skeletal malformations and hypotonia					
20	c.4723C > T/c.5053C > T	p.R1575*/p.R1685*	AR	Bangladeshi	Chesler (2016) N Engl J Med 375, 1355 [25]
21	c.5054G > C/c.5053C > T	p.R1685P/p.R1685*	AR	Mixed European and Japanese	Chesler (2016) N Engl J Med 375, 1355 [25]
Muscular atrophy with perinatal respiratory distress, arthrogryposis & scoliosis					
22	c.3020_3030del/c.3020_3030del	p.P1007Lfs*3/ p.P1007Lfs*3	AR	India	Vedove (2016) Am J Hum Genet 99, 1206 [21]
23	c.5621delT/ c.5621delT	p.L1874Rfs*5/ p.L1874Rfs*5	AR	Turkey	Vedove (2016) Am J Hum Genet 99, 1206 [21]
24	c.1550_1552delGCTinsCGAA/c.1550_1552delGCTinsCGAA	p.S517Tfs*48/ p.S517Tfs*48	AR	Libya	Vedove (2016) Am J Hum Genet 99, 1206 [21]
25	c.493-?_917 +?del/c.493-?_917 +?del		AR	Pakistan	Vedove (2016) Am J Hum Genet 99, 1206 [21]
Arthrogryposis, distal with muscle weakness, scoliosis & proprioception defects					
26	c.1384C > T/c.1384C > T	p.R462*/p.R462*	AR	Turkey	Haliloglu (2017) J Hum Genet 62, 497 [26]
Short stature, scoliosis, gross motor impairment, progressive contractures, and loss of proprioception and touch sensation					
27	c.2708C > G/c.2708C > G	p.S903*/p.S903*	AR	Bangladesh	Mahmud (2017) Clin Genet 91, 470 [27]

*NA* information not available

## Discussion

DA is defined broadly as a heterogeneous category of inherited limb malformation syndromes with substantial clinical and genetic heterogeneity and variable expressivity [3, 12, 13]. Here, we report two novel mutations within *TPM2* and *PIEZO2* responsible for DA2B and mild DA in two Chinese families, respectively.

Mutations in *TPM2* were previously thought to solely cause DA1 [13]. However, mutations in *TPM2* have recently been reported to be associated with DA2B [14, 15]. In line herewith, our patients carrying a missense mutation p.Q103R in *TPM2* presented typical DA2B as well. Our study supports that DA1 and DA2B may be categorized as phenotypic extremes of the same disorder rather than as different DA types [15]. Currently, HGMD (Professional 2018.1) shows that 35 disease-causing mutations within *TPM2* cause 11 different myopathic conditions including nemaline myopathy, congenital myopathy, DA2B, DA1 and Cap disease [16–19]. The identified mutations were distributed across the *TPM2* gene and cannot be mapped to any hotspots. The number of mutations related to DA1 and DA2B account for 25.7% (9/35) of all reported, most of which are missense mutations (8/9) (Fig. 3e). According to the mutation of *TPM2* in this study and retrospective analysis of related studies, we found that there is not any obvious correlation between types or location of *TPM2* mutations and clinical manifestations (Fig. 3e), as reported by Beck et al. and Marttila et al. [15, 20].

Mutations in *PIEZO2* have been reported in Marden-Walker syndrome, DA3, DA5, and other DAs that involve in sense of touch damage and impaired proprioception. Piezo2 is a transmembrane protein, which can be found in many tissues, with a function of adapting mechanically activated currents. It composes of stretch-activated ion channels, and was reported to be associated with touch sensation [21]. Our study presents Chinese patients carrying a missense mutation p.R2718Q in *PIEZO2* for the first time who have mild arthrogryposis without other manifestations. The mutation c.8153G > T (p.R2718L) in *PIEZO2* was previously described in a family with a subtype of DA5 characterized by contractures, limited eye movements, restrictive lung disease, and variably absent cruciate knee ligaments [8]. McMillin et al. reported a male patient with DA5 in whom a heterozygous mutation c.8153G > C was identified in *PIEZO2*. This mutation yielded an arginine-to-proline (R2718P) substitution and induced the features of ptosis, ophthalmoplegia, scoliosis, and pulmonary disease [8]. Although the mutation site is the same for these two cases and the patients in this study, the substituted amino acid differs, and resulted various phenotypes, which may enlighten the association between genotypes and phenotypes. HGMD (Professional 2018.1) records 26 disease-causing mutations in *PIEZO2* and 10 resulted disorders, and the DA5 is the most common condition (14/26) in *PIEZO2*-related disorders (Table 3) (Fig. 4d). Heterozygous gain-of-function (GoF) mutations in *PIEZO2* have been reported in MWKS, DA3, DA5, which typically involve congenital contractures of hands and feet, or cleft palate, ophthalmoplegia, ptosis, and cerebellar malformations [8, 22–24]. Recent studies have discovered that biallelic loss-of-function (LoF) mutations in *PIEZO2* cause muscular atrophy with perinatal respiratory distress, arthrogryposis, scoliosis and proprioception defects [21, 25–27]. Phenotype-genotype correlations analysis suggests that heterozygous mutations carried by the DA3, DA5, MWKS patients are mainly localized at the C-terminal of PIEZO2, which results in increased channel activity*.* Nevertheless, all of the recessive inherited *PIEZO2*-related disorders are caused by biallelic truncating mutations which may induce nonsense-mediated decay of the mRNA [21] (Table 3) (Fig. 4d). As GoF and LoF variants in *PIEZO2* have different effects on muscle development and disease pathophysiology, different inheritance modes and mutation types lead to distinct DA phenotypes.

## Conclusions

In conclusion, we firstly reported two novel mutations within *TPM2* and *PIEZO2* causing DA2B and mild DA in two Chinese families, respectively. Our study also expands the spectrum of causal mutations in the *TPM2* and *PIEZO2* genes.

## Additional files

Additional file 1: **Table S1.** Primer used in this study (XLS 10 kb)

Additional file 2: **Figure S1.** Ultrasound examination of a fetus (IV: 1) at 22 weeks of gestational age. (A) Arrow indicates extended wrist and clenched hand. Polyhydramnios is apparent. (B) Arrow specifies bilateral clubfoot with a deformity of the toes (PPT 329 kb)

Additional file 3: **Table S2.** Two-point linkage analysis using 3 genetic markers in the DA2B family (XLSX 9 kb)

## Abbreviations

DA: Distal arthrogryposis
HGMD: Human Gene Mutation Database
LOD: Logarithm of odds
PCR-RFLP: Polymerase chain reaction–restriction fragment length polymorphism
PROVEAN: Protein variation effect analyzer
SIFT: Scale-invariant feature transform
WES: Whole-exome sequencing
