# Supplementary figures and images for: Novel mutations in TPM2 and PIEZO2 are responsible for distal arthrogryposis (DA) 2B and mild DA in two Chinese families
Source: BMC Med Genet. 2018 Oct 3;19:179. doi: 10.1186/s12881-018-0692-8 (PMC6171138; doi:10.1186/s12881-018-0692-8)

## Slide 1
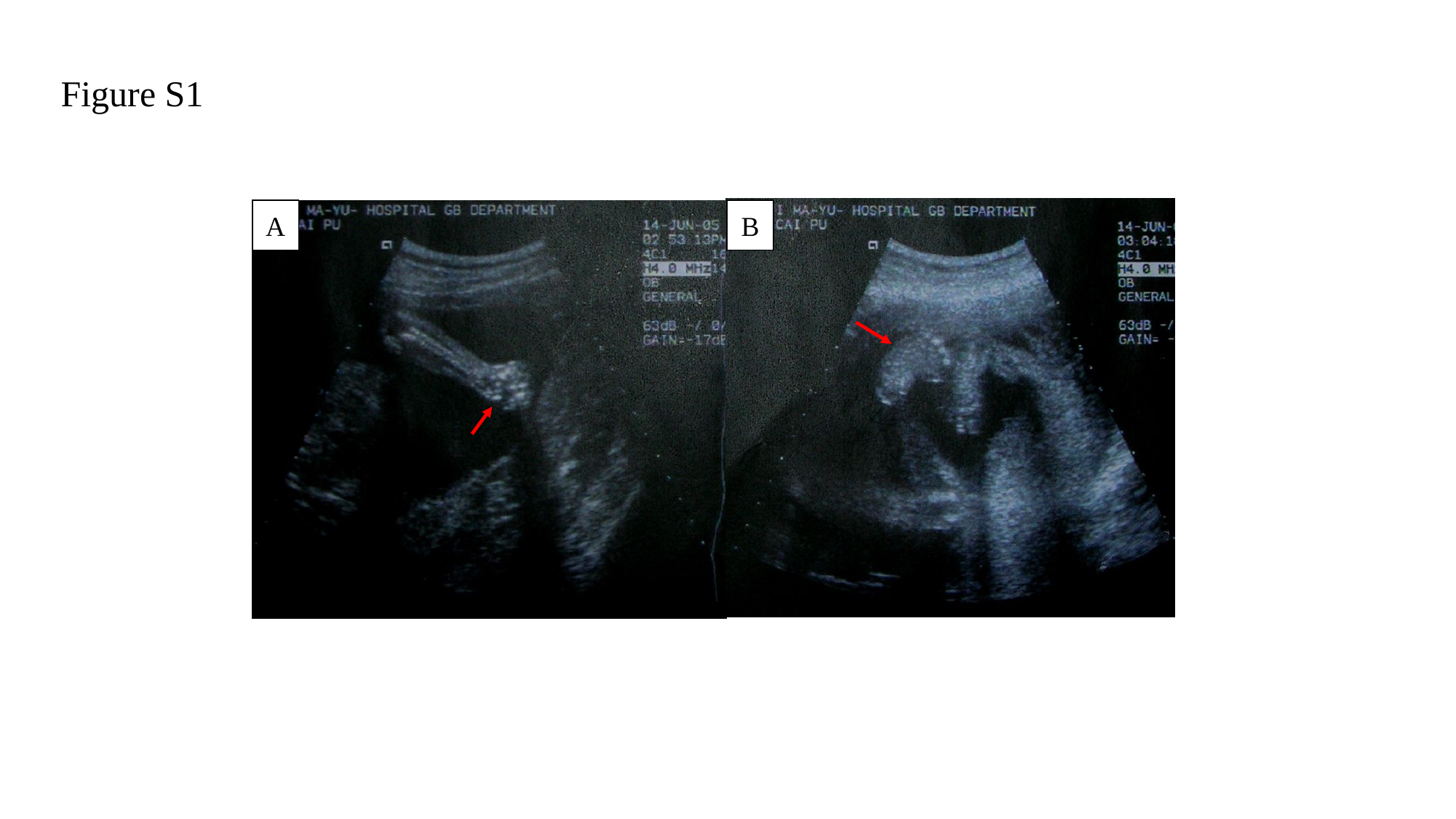

Figure S1
A
B

Supplement: Supplementary file 2 — Figure S1. Ultrasound examination of a fetus (IV: 1) at 22 weeks of gestational age. (A) Arrow indicates extended wrist and clenched hand. Polyhydramnios is apparent. (B) Arrow specifies bilateral clubfoot with a deformity of the toes (PPT 329 kb) [file 12881_2018_692_MOESM2_ESM.ppt]
